# Decolorized and Undecolorized Ethanol Extracts of Ginggiyang (*Leea aequata* L.) Leaves as Effective Preservatives of Edible Products

**DOI:** 10.17113/ftb.63.04.25.8838

**Published:** 2025-12-26

**Authors:** Purwaniati Purwaniati, Rahmana Emran Kartasasmita, Maria Immaculata Iwo, Muhamad Insanu

**Affiliations:** 1 Department of Pharmaceutical Chemistry, School of Pharmacy, Bandung Institute of Technology, Bandung, Indonesia; 2 Department of Pharmaceutical Analysis and Medicinal Chemistry, Pharmacy Faculty, Bhakti Kencana University, Bandung, Indonesia; 3 Department of Pharmaceutical Biology, School of Pharmacy, Bandung Institute of Technology, Bandung, Indonesia; 4 Department of Pharmacology and Clinical Pharmacy, School of Pharmacy, Bandung Institute of Technology, Bandung, Indonesia

**Keywords:** hedonic test, *Leea aequata* L., natural preservatives, total plate count, yeast and mould count

## Abstract

**Research background:**

The global increase in population has led to a corresponding rise in the production of edible products and chemical preservatives. However, chemical preservatives are often associated with adverse health effects, highlighting the need to develop safer and more effective alternatives.

**Experimental approach:**

This research evaluates the effectiveness and sensory acceptability of decolorized and undecolorized ethanol extracts of *Leea aequata* L. leaves as preservatives. The assessment involved applying the extracts to bread, jam and juice products, and conducting preliminary hedonic tests of bread and juice samples to determine sensory acceptability. The total plate count (TPC) and yeast and mould (YM) count were used to evaluate the effectiveness of preservation. Comparisons were made with a control without added preservatives and with products preserved using potassium sorbate.

**Results and conclusions:**

Products treated with test extracts were as sensorially acceptable as those treated with potassium sorbate. The addition of the extracts was shown to have a preservative effect, as the TPC and YM counts were lower than in the control. Therefore, the test extracts have the potential to be developed as natural preservatives that could replace chemical variants such as potassium sorbate.

**Novelty and scientific contribution:**

Decolorized and undecolorized ethanol extracts of *Leea aequata* L. leaves were shown for the first time as preservatives of edible products. The results of these tests are crucial for developing effective preservatives.

## INTRODUCTION

The continuous growth of the global population has led to an increasing demand for food products. Consequently, innovations in food production must be accompanied by the development of new additives, including preservatives. In this context, the pursuit of safer, more effective and sustainable preservative alternatives has become a pressing priority (*1*–*4*). The demand for the discovery of natural preservatives, specifically those derived from plants, is also increasing (*5*, *6*).

According to previous research, microbial contamination of food is the main reason for the need for preservatives (*^(^**^7^**^)^*). This contamination can cause product spoilage and various foodborne diseases. The World Health Organization (WHO) states that 600 million people fall ill from foodborne pathogens each year (*8*). Microbial contamination can lead to a range of foodborne illnesses, with symptoms varying from mild discomfort to life-threatening conditions (*9*). These conditions are generally caused by contamination of food products with foodborne pathogens, such as bacteria, fungi, viruses and parasites (*10*). Typical examples of pathogenic bacteria include *Bacillus cereus*, *Campylobacter jejuni*, *Clostridium botulinum*, *C. perfringens*, *Cronobacter sakazakii*, *Escherichia coli*, *Listeria monocytogenes*, *Salmonella* spp., *Shigella* spp., *Staphylococcus aureus*, *Vibrio* spp. and *Yersinia enterocolitica*. These microbes can be found in various forms and have different properties. Some bacteria, including *C. botulinum* (*11*), *C. perfringens*, *B. subtilis* and *B. cereus* (*12*), can form heat-resistant spores. These bacteria can survive even when exposed to high temperatures. *S. aureus* and *C. botulinum* are also able to produce heat-resistant toxins (*11*, *13*). Meanwhile, pathogens such as *L. monocytogenes* and *S. enterica* can grow in cold temperatures (*14*). The properties of these pathogenic bacteria present challenges in maintaining the quality of edible products, as some have the potential to evolve to become heat-resistant (*15*). This adaptation renders preservation technologies that rely solely on heat treatment ineffective, underscoring the need to develop new preservatives.

Globally, an estimated 300 fungal species pose health risks, the majority of which are foodborne pathogens (*16*). Several fungi excrete mycotoxins, which contaminate food and cause acute poisoning, cancer, liver disease and other illnesses (*17*). *Aspergillus*, *Candida*, *Alternaria* and *Fusarium* are fungal genera that frequently become foodborne pathogens (*18*, *19*). Hepatitis A and norovirus are among the group of pathogenic viruses, while *Cyclospora cayetanensis*, *Toxoplasma gondii* and *Trichinella spiralis* are classified as parasitic pathogens (*10*).

Based on the results, preservatives commonly used in edible products in the community are chemicals added to food to slow down deterioration. Preservatives are additives permitted in food products, but their use must be regulated to ensure safety, and they should be produced in accordance with good manufacturing practices. Moreover, their addition must not exceed the required limit as stipulated in the regulations and must be food-grade (*20*–*22*). The most commonly used preservatives are weak acid compounds and their salts, such as sorbic acid (*23*, *24*) and benzoic acid (*25*). Although the compounds are included in the GRAS (generally recognized as safe) category (*24*), continuous long-term use can lead to resistance (*25*), which further increases the need to discover new preservatives.

Therefore, this research aims to evaluate the effectiveness and acceptability of ethanol extracts of decolorized and undecolorized *Leea aequata* leaves in preserving edible product samples. The potential of this extract as a preservative was first indicated by a previously published antimicrobial activity test (*26*), which demonstrated that this extract has antimicrobial activity against several foodborne pathogens, both bacteria and fungi. Microbes that are sensitive to this extract include *Candida albicans*, *B. subtilis*, *S. aureus*, *E. coli*, *P. aeruginosa* and *Aspergillus flavus* (*26*). Its effectiveness as a preservative was then tested on food samples by determining the total plate count (TPC) and yeast and mould count (YM), and acceptability was assessed using a hedonic test.

## MATERIALS AND METHODS

The 96 % ethanol (Merck, Darmstadt, Germany), powdered activated carbon (PAC) (Merck, Darmstadt, Germany), potassium sorbate (Jiangsu Mupro IFT Corp., Lianyungang, Jiangsu Province, PR China), Avicel 102 (Dupon Pharma, Wilmington, NC, USA), demineralized water (Bratachem, Bandung, Indonesia), 3M™ Petrifilm™ yeast and mould count plate (Petrifilm YM) and 3M™ Petrifilm™ aerobic count plate (Petrifilm AC) (3M, Saint Paul, MN, USA) and buffered peptone solution (Merck, Darmstadt, Germany) were used in the research. The ingredients for making bread samples were: flour (Indofood Sukses Makmur, Jakarta, Indonesia), sugar (Sugar Group Companies, Lampung, Indonesia), instant yeast (Lesaffre, Marcq-en-Baroeul, France), milk powder (Nestle, Pasuruan, Indonesia), cocoa powder (BT Cocoa, Tangerang, Indonesia), margarine (Upfield Manufacturing Indonesia, Bekasi, Indonesia), chicken eggs, and salt (Refina, Surabaya, Indonesia). Ingredients for making jam (pineapple, sugar and salt), and juice (guava fruit and sugar) were obtained from local supermarkets in the city of Bandung, Indonesia.

### Plant extract preparation

The test extracts included decolorized and undecolorized ethanol extracts. The extracts were prepared by maceration, where 1 kg of simplicia powder from *Leea aequata* leaves was macerated with 3×5 L of 96 % ethanol. The solvent was replaced every 24 h, and the extracts were collected, filtered and then concentrated using a rotary evaporator (RV-8; IKA, Staufen im Breisgau, Germany) until a viscous extract was obtained. The thick extract was dried with a small amount of microcrystalline cellulose (Avicel 102) to accelerate the drying process in an oven (UF260; Memmert, Schwabah, Germany) at 40 °C until constant mass. Then the extract was mixed with Avicel 102 until a final *m*(dry extract):*m*(Avicel 102)=2:3 was obtained. Decolorized extract was prepared using the same maceration method as for the undecolorized extract. After filtration, the extract was decolorized by adding 2 g of PAC to every 100 mL of extract. The extracts were stirred using a magnetic stirrer (RCT Basic; IKA) for 1 h, filtered, and concentrated using a rotary evaporator until a thick extract was obtained, which was dried at 40 °C in an oven with a small amount of Avicel 102 and further mixed until the final ratio of the two substances was 1:1.

### Addition of extracts to bread samples

Each 1200 g batch of bread dough was prepared using high protein flour (500 g), granulated sugar (150 g), instant yeast (11 g), milk powder (52 g), cocoa powder (20 g), one chicken egg, water (260 g), margarine (100 g), salt (5 g) and preservatives. Six formulations were made, differing in the type and amount of preservatives used: bread A contained no added preservatives, bread B contained 1500 mg decolorized extract, bread C contained 2500 mg decolorized extract, bread D contained 1500 mg undecolorized extract, bread E contained 2500 mg undecolorized extract, and bread F contained 1000 mg potassium sorbate as a comparative preservative. The dough was shaped into loaves of approx. 14 g and baked at 200 °C for 30 min. The bread loaves were individually packaged in an oriented polypropylene (OPP) laminated bag, size 6 cm×12 cm (Indah Karunia Sukses, Jakarta, Indonesia), and stored at room temperature ((25±2) °C) for 12 days until analysis was completed.

### Addition of extracts to pineapple jam samples

Jam was made by mixing grated pineapple (1 kg), sugar (100 g), salt (5 g) and preservatives. Jam A contained no preservatives, while jams B, C and D contained 1000 mg decolorized extract, 1000 mg undecolorized extract and 1000 mg of potassium sorbate as a comparative preservative, respectively. Each mixture was then cooked over medium heat for 60 min. The product was packaged in plastic cups (Wingoh Albindo, Tangerang, Indonesia), with 10 g per cup, and stored at room temperature ((25±2) °C) for 12 days until analysis was completed.

### Addition of extracts to guava juice samples

Guava juice was prepared by blending 250 g of fresh guava, 50 g of sugar, 500 mL of water, and several preservatives: juice A contained no preservatives, while juices B, C and D contained 750 mg decolorized extract, 750 mg undecolorized extract and 1000 mg of potassium sorbate as a comparative preservative, respectively. The juices were packaged in plastic bottles (Indo Tirta Abadi, Tangerang, Indonesia), 50 mL per bottle, and stored in a refrigerator (SCH-405X-WH3; Sharp, Karawang, Indonesia) at (4±2) °C for 12 days until the analysis was completed.

### Organoleptic observation and hedonic test

Organoleptic observations (colour, odour, texture, and presence or absence of fungal growth) were made on days 1, 3, 5 and 7, with taste evaluated on the first day.

The hedonic test was conducted with 40 untrained panellists, who were not informed about the ingredient composition of each food product. Each panellist evaluated six bread samples and four juice samples, and no incentives were offered for participation. The panellists were university students in Bandung, aged 18–23, consisting of 20 men and 20 women. The panellists had an average ability to distinguish and communicate organoleptic differences (colour, taste, texture and aroma) of the food samples tested. The participants were asked to give a general preference score using a 9-point hedonic scale: 1=dislike extremely, 2=dislike very much, 3=dislike, 4=dislike slightly, 5=neither like nor dislike, 6=like slightly, 7=like, 8=like very much, and 9=like extremely (*27*). At each sampling session, panellists were first asked to clean their mouths (gargle) with mineral water, observe the colour, smell, taste and texture of each sample and assign an overall acceptance score.

### Determination of yeast and mould count in food product samples

The determination was carried out using Petrifilm YM medium, according to the manufacturer’s instructions. A total of 10 g of sample was placed in a 100-millilitre glass beaker and then 0.1 % buffered sterile peptone water was added. The sample was homogenised by sonication for 10 min. The sample solution was diluted to obtain dilutions of 10, 100 and 1000 times, which were expected to yield yeast and mould counts in the range of 25–250. The cover was removed from the Petrifilm YM plate, which was placed on a flat surface in the biosafety cabinet, and 1 mL of each dilution was dripped on the centre of the plate. The cover was then replaced, and a sample droplet was placed on the top right side and pressed using a spreader. The sample was subsequently spread in a circular motion to match the diameter of the spreader. The Petrifilm YM plates were then incubated at 25 °C for 5 days with the cover on top, with no more than 20 plates stacked. The plates were removed from the incubator on the fifth day, and the number of yeast and mould colonies was counted. Yeast colonies were small, three-dimensional, uniform in size and pale pink to turquoise in colour. In contrast, mould colonies were large, wide, flat, diverse in colour, with a dark centre. ^The number of y^easts and moulds ^was determined at various times^, on days 1, 3, 5 and 7.

### Determination of total plate count in food product samples

The total plate count in food product samples was determined using Petrifilm AC plates. A mass of 10 g of sample was placed in a 100-millilitre glass beaker and then 0.1 % buffered sterile peptone solution was added. The sample was homogenised by sonication for 10 min. The sample solution was diluted 10, 100 and 1000 times, aiming to yield a total plate count between 25 and 250. The cover of the Petrifilm AC plate was removed and the plate was placed on a flat surface in the biosafety cabinet. The centre was inoculated with 1 mL of the sample at each dilution. The cover was then closed, and a sample droplet was placed on its right side and pressed using a spreader. The sample was then spread in a circular motion to match the diameter of the spreader. The Petrifilm AC plates were incubated for 72 h at 30 °C. Afterwards, the plates were removed from the incubator and the number of colonies was counted. The determination was carried out at different time points, specifically on days 1, 3, 5 and 7.

### Statistical analysis

The results were expressed as the mean value±standard deviation of at least three independent tests. These research data were statistically analysed following an initial normality test. Data normality was tested using the Anderson-Darling method (*28*). Normally distributed data were analysed using one-way ANOVA, while non-normally distributed data were analysed using the Kruskal–Wallis test, followed by Tukey’s or Dunn’s *post hoc* test (*29*). Statistical analysis was carried out using Minitab v. 21.1 software (*30*), with a significance level of p<0.05.

## RESULTS AND DISCUSSION

The addition of the test extract and standard preservative (potassium sorbate) caused organoleptically observable differences in the resulting food products (Table 1). Food samples preserved with decolorized extract had almost the same texture, colour and aroma as products that were either not preserved or preserved with potassium sorbate. In contrast, food samples preserved with undecolorized extract had different textures, colours and aromas. This effect is attributed to the high pigment content of the undecolorized extract, particularly chlorophyll, which is the primary pigment in leaves that plays a role in photosynthesis (*31*). Prolonged exposure to light causes degradation and bleaching of the chlorophyll (*32*). The cooking process at high temperatures, such as bread baking and jam preparation, also accelerates chlorophyll damage, resulting in changes to food texture and taste. Food products preserved with undecolorized extract are reported to have unpleasant colour, texture and taste (*33*). In the decolorized extract, most of the pigments have been removed, so the effects of chlorophyll were minimal.

**Table 1:** Organoleptic observations of bread, jam and juice samples without preservatives, with decolorized and undecolorized ethanol extracts and potassium sorbate during storage

Sample	*t*(storage)/day				
1	3	5	7	12
Bread A	Bread rises, characteristic aroma of bread, brown colour	Same as day 1	Mould growth began to appear	Mould is spreading	The mould covers almost the entire surface of the bread
Bread B	Bread rises, characteristic aroma of bread, brown colour	Same as day 1	Same as day 1	Colour fades	Less mould growth than on bread A
Bread C	Bread rises, characteristic aroma of bread, brown colour	Same as day 1	Same as day 1	Colour fades	Less mould growth than on bread B
Bread D	The bread is very fluffy in the oven, but it deflates after cooling. The surface of the bread wrinkles, and there is a characteristic aroma of the extract, a slightly greenish-brown colour	Same as day 1	Same as day 1	Colour fades	The mould growth is almost the same as in bread C
Bread E	The bread is very fluffy in the oven, but it deflates after cooling. The surface of the bread wrinkles, and there is a characteristic aroma of the extract, a slightly greenish-brown colour	Same as day 1	Same as day 1	Colour fades	More mould growth than on bread D
Bread F	Bread is less fluffy than bread loaves A–E and has a denser texture, brown colour and distinctive aroma	Same as day 1	Same as day 1	Same as day 1	Same as day 1
Jam A	Distinctive yellow colour of the pineapple, sharp taste and distinctive aroma	Same as day 1	Growing mould	Growing mould	Increased mould growth
Jam B	The yellow colour is typical of pineapple, and the typical aroma of pineapple is weak	Same as day 1	Same as day 1	Growing mould	Increased mould growth
Jam C	Greenish-yellow colour, weak pineapple aroma, dominant extract aroma	Same as day 1	Same as day 1	Growing mould	Increased mould growth
Jam D	The distinctive yellow colour of the pineapple and its distinctive aroma	Same as day 1	Same as day 1	Same as day 1	Same as day 1
Juice A	Bright pink colour, more stable juice dispersion, signature aroma of guava juice	Same as day 1	Same as day 1	Formed two layers	Built-in 2 layers
Juice B	Faded pink colour, foamy, unstable juice dispersion, and a characteristic aroma of guava juice	Same as day 1	Juice separates into two layers	Juice separates into two layers	Juice separates into two layers
Juice C	Faded pink colour, foamy, unstable juice dispersion, and a characteristic aroma of guava juice	Same as day 1	Same as day 1	Same as day 1	Same as day 1
Juice D	Pink colour, less stable juice dispersion	Juice separates into two layers	Juice separates into two layers	Juice separates into two layers	Juice separates into two layers

Bread samples A–F=formulations without preservative, with 1500 mg decolorized extract, with 2500 mg decolorized extract, with 1500 mg undecolorized extract, with 2500 mg undecolorized extract, and with 1000 mg potassium sorbate, respectively; jam samples A–D=formulations without preservative, with 1000 mg decolorized extract, with 1000 mg undecolorized extract, and with 1000 mg potassium sorbate, respectively; and juice samples A–D=formulations without preservative, with 750 mg decolorized extract, with 750 mg undecolorized extract, and with 1000 mg potassium sorbate, respectively

Samples of bread and jam preserved with decolorized and undecolorized extracts maintained their quality until the 5th day of observation. Physical changes were observed only on day 7, and mould growth began to appear on day 12 in preserved bread samples and on day 7 in preserved jam samples, in contrast to bread and jam without preservatives, where mould growth started from day 3. This confirmed that the addition of decolorized and undecolorized extracts can maintain the quality of bread and jam for up to five days. However, bread and jam preserved with undecolorized extract had poor colour and aroma. Organoleptic tests showed that decolorized extract inhibited the growth of microbes in bread and jam samples, without affecting their colour, taste and texture. In jam samples, adding decolorized and undecolorized extracts was less effective than using potassium sorbate. The same trend was observed in samples with and without decolorized and undecolorized extracts.

The preservative effects of decolorized and undecolorized extracts are attributed to the antimicrobial properties of the plant extract. *Leea aequata* has been proven to contain several antimicrobial compounds, such as scopoletin, vanillic acid and kaempferol (*34*). Scopoletin has antibacterial (*35*–*37*), antifungal (*38*), antiparasitic and even antiviral properties (*39*), while kaempferol has antibacterial (*40*) and antifungal (*41*) properties. Vanillic acid has been shown to have broad-spectrum antibacterial activity (*42*).

The hedonic test measures the level of consumer preference for a food product. This is a crucial test in the development of every food product. The 9-point hedonic scale has been rated the best for over 60 years, although other, more superficial scales have been developed (*27*). A preliminary hedonic test of bread and juice samples was performed as part of sensory quality measurements (*43*) to determine the overall preferences of food products with decolorized or undecolorized extracts. The addition of decolorized or undecolorized ethanol extract to the tested food products significantly affected the preference compared to a product with no added preservatives. However, it was not significantly different from products preserved with potassium sorbate.

Differences in panellist preferences for bread and juice products preserved with test extracts were evaluated using the Kruskal–Wallis test, as the data were not normally distributed (*29*). This test revealed a difference in the panellist preferences for the tested samples, and a *post hoc* analysis was used to determine which test groups differed significantly.

**Fig. 1:**
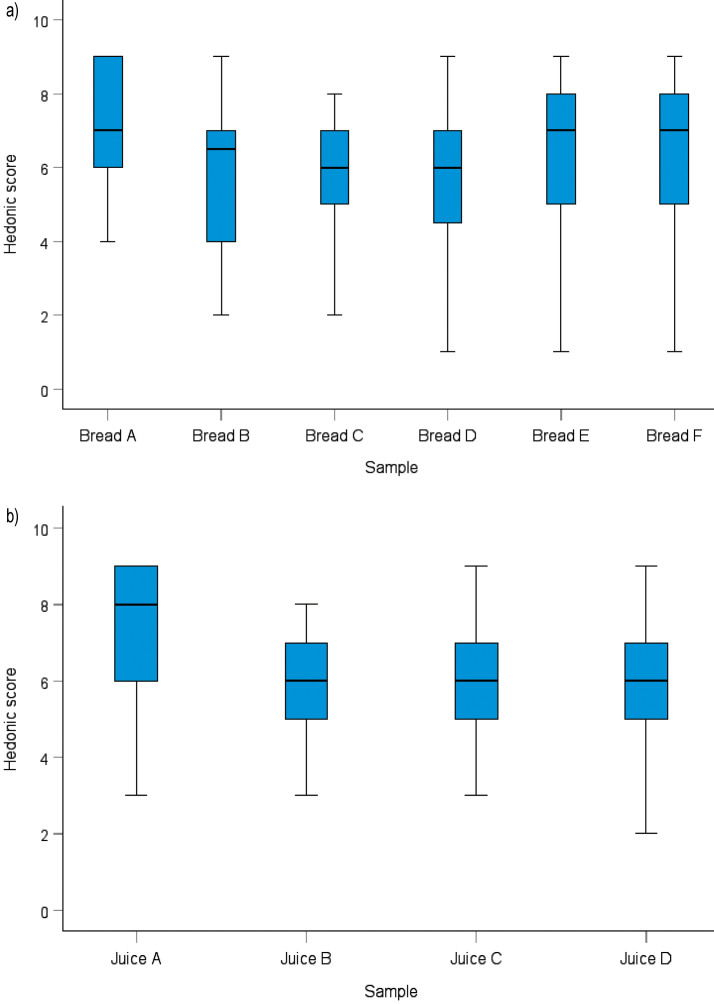
Boxplot of preliminary hedonic scores for: a) bread, and b) juice samples with different preservative treatments. For bread, sample codes are: A=control without preservatives, B and C=decolorized ethanol extracts, D and E=undecolorized ethanol extracts, and F=potassium sorbate. For juice, sample codes are: A=control, B=decolorized extract, C=undecolorized extract, and D=potassium sorbate. Higher scores indicate greater panellist acceptance

The addition of plant extracts as preservatives to bread and juice samples resulted in a decrease in overall preferences (colour, taste, texture and aroma) compared to unpreserved products (p<0.05). However, there was no difference in preferences between the food products containing the tested extracts and those containing the standard preservative (potassium sorbate). This suggests that the plant extracts could substitute the commonly used potassium sorbate (Fig. 1).

Total plate count and yeast and mould plate count are essential indicators in the food industry. These parameters can be used to predict the sanitary quality and shelf life stability of food products (*44*). They can also serve as indicators of microbial contamination (bacteria or yeasts and moulds, respectively) of food products, poor product processing or inadequate storage facilities (*45*). The total plate count is also known as the standard plate count, aerobic mesophilic count, aerobic plate count and aerobic colony count (*45*).

**Fig. 2:**
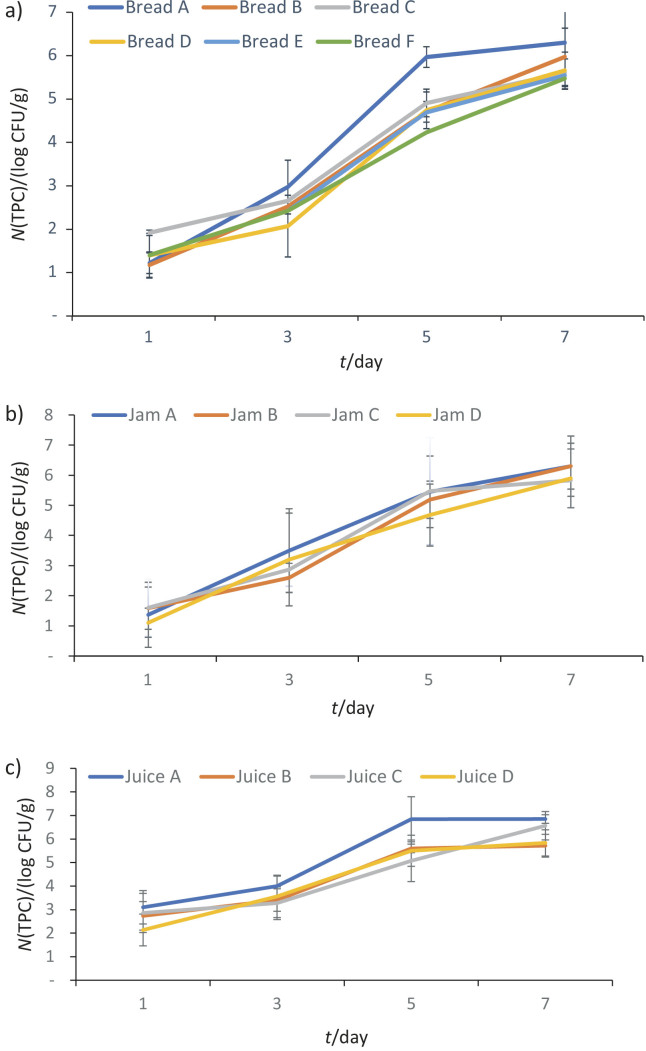
Total plate count (TPC) in: a) bread, b) jam, and c) juice samples during storage. Bread samples: A=without preservatives, B and C=decolorized ethanol extract, D and E=undecolorized ethanol extract, and F=potassium sorbate, jam and juice samples: A=without preservatives, B=decolorized extract, C=undecolorized extract, and D=potassium sorbate. Values are expressed as mean±standard deviation

Fig. 2 shows that the total plate count of bread preserved with decolorized extract, undecolorized extract, or potassium sorbate was significantly lower than that of the unpreserved products. The total plate count values of samples without preservatives (bread A and juice A) increased significantly compared to those with preservatives (p<0.001). This finding suggests that decolorized extract, undecolorized extract and potassium sorbate effectively inhibited the growth of aerobic bacteria during storage. This pattern aligns with research conducted by Atwaa *et al*. (*4*), who observed a reduction in microbial load in tomato paste and pasteurized milk products preserved with various plant extracts, such as fenugreek seed, sage leaf, thyme leaf, rosemary leaf and clove extracts.

The antimicrobial effects are thought to be linked to phenolic and flavonoid compounds in these extracts, which disrupt the bacterial cell membrane wall and interfere with their metabolic enzymes (*3*, *40*, *41*). A previous study has shown that the leaves of *Leea aequata* contain various phenolic and flavonoid compounds (*46*), which supports this hypothesis.

**Fig. 3:**
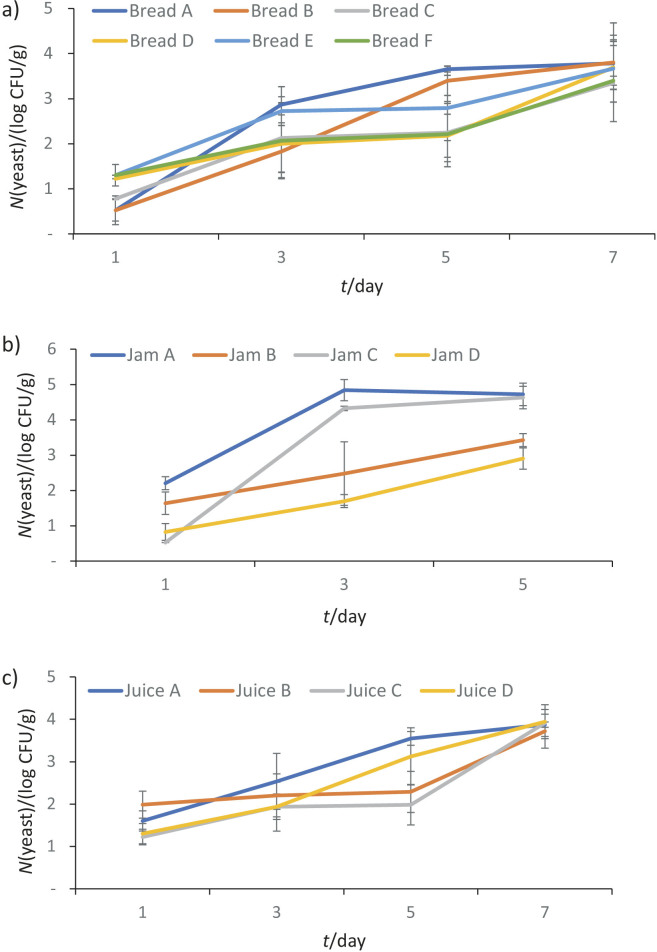
Yeast count in: a) bread, b) jam, and c) juice samples during storage. Bread samples: A=without preservatives, B and C=decolorized ethanol extract, D and E=undecolorized ethanol extract, and F=potassium sorbate, jam and juice samples: A=without preservatives, B=decolorized extract, C=undecolorized extract, and D=potassium sorbate. Values are expressed as mean±standard deviation

**Fig. 4:**
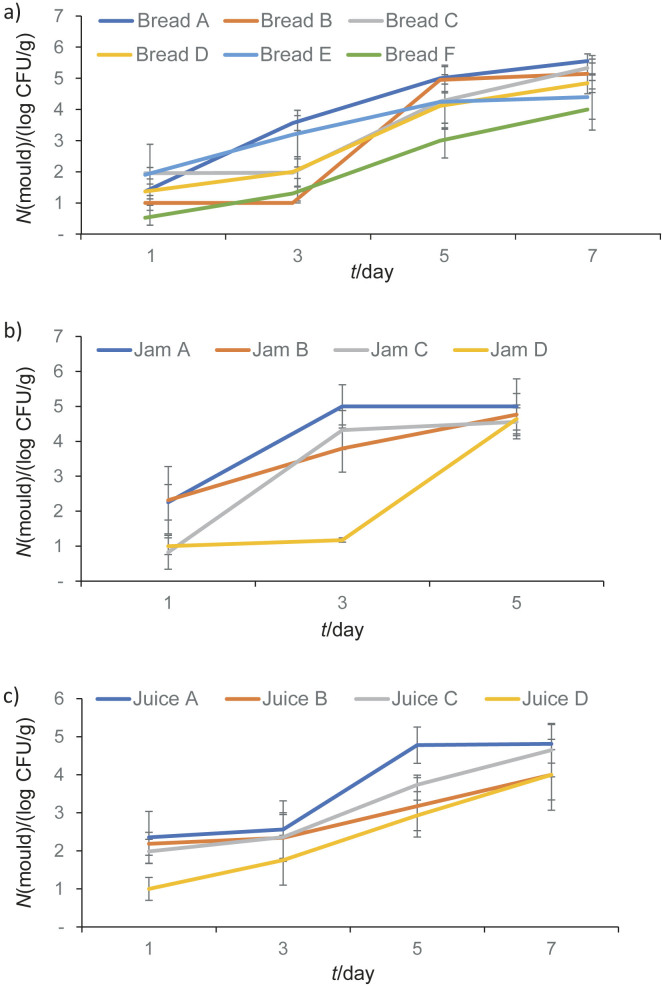
Mould count in: a) bread, b) jam, and c) juice samples during storage. Bread samples: A=without preservatives, B and C=decolorized ethanol extract, D and E=undecolorized ethanol extract, and F=potassium sorbate, jam and juice samples: A=without preservatives, B=decolorized extract, C=undecolorized extract, and D=potassium sorbate. Values are expressed as mean±standard deviation

Fig. 3 shows that the addition of decolorized extract, undecolorized extract or potassium sorbate to bread, juice and jam samples caused a significant decrease in yeast count compared to unpreserved products (p<0.001). Similarly, Fig. 4 indicates that food products preserved with decolorized extract, undecolorized extract or potassium sorbate had significantly lower mould counts than unpreserved products. This clearly demonstrates the antifungal properties of decolorized extract, undecolorized extract and potassium sorbate. The prevention of yeast and mould growth in food products by adding sage, rosemary and clove extracts has been previously studied (*4*). This research adds to the extensive list of plants with potential as preservatives.

Both decolorized and undecolorized ethanol extracts inhibited the growth of bacteria and fungi in bread and juice samples, with no significant differences observed (p>0.05). In the jam samples, the YM count was 10^5^ CFU/g on day 5 of measurement, so the measurement on day 7 was not carried out.

Using natural ingredients as preservatives has a long history in food preservation. However, to date, no approvals have been granted by the Joint FAO/WHO Expert Committee on Food Additives (JECFA) for any natural preservative. Bread is frequently examined in studies assessing preservation effectiveness, primarily because it is essential to preserve bread, particularly when stored at room temperature (*47*). Natural ingredients proven effective in preserving bread include raisin extract (*48*), mustard bran and flour (*49*), oregano (*Origanum vulgare* L.) essential oil (*50*) and rosemary (*Rosmarinus officinalis* L.) essential oil (*51*). Although rosemary essential oil is effective in preserving food samples, its official use as a preservative has not yet been approved by the JECFA, unlike its extract, which has been approved for use as an antioxidant (*51*). The abundant potential of natural materials as preservatives, none of which has been approved for international use, remains an interesting challenge in research.

## CONCLUSIONS

Decolorized and undecolorized ethanol extracts of *Leea aequata* L. leaves were tested for their preservative effects in bread, jam and juice products. Both extracts inhibited the growth of microorganisms (bacteria and fungi), especially in bread and juice products. The decolorized extract showed a more robust and sensorially acceptable antimicrobial effect, indicating significant potential for further development as a viable natural preservative for edible products. The novelty of this work lies in the development, evaluation and diversification of the application of *Leea aequata* leaves, extending beyond their traditional use as preservatives for palm sap. This research is highly relevant for the development of safe and effective plant-based preservatives, providing a promising foundation for further standardization, safety evaluation, and industrial application of *Leea aequata* extracts.
